# Sarcoidosis With Skeletal Involvement Masquerading as Metastatic Malignancy

**DOI:** 10.7759/cureus.44457

**Published:** 2023-08-31

**Authors:** Arthur M Samia, Stephanie Fabara Pino, Liang Sun

**Affiliations:** 1 Dermatology, University of Florida, Gainesville, USA; 2 Internal Medicine, University of Central Florida, School of Medicine/North Florida Hospital, Gainesville, USA

**Keywords:** b-symptoms, immunoelectrophoresis, non-caseating granuloma, necrotizing sarcoid granulomatosis, bone sarcoidosis, neurosarcoidosis

## Abstract

Sarcoidosis is a systemic disorder characterized by noncaseating granuloma formation, which can affect any organ in the body; however, skeletal involvement is relatively uncommon. This case report presents a rare case of sarcoidosis primarily affecting the skeletal system in a 39-year-old man with a history of neurosarcoidosis. The patient presented with symptoms of nausea, vomiting, fatigue, weight loss, and lower back and pelvic pain, which were initially suspicious for malignancy. Computed tomography scans revealed lytic bone lesions and lymphadenopathy. However, a biopsy of a left inguinal lymph node confirmed necrotizing granulomatous lymphadenitis, which was consistent with necrotizing sarcoid granulomatosis - a rare variant of sarcoidosis. The patient was treated with systemic corticosteroids, which led to clinical improvement. The prognosis of sarcoidosis is generally good, with spontaneous remission occurring in up to two-thirds of patients; however, some patients may develop chronic and/or progressive disease. In particular, patients with a history of neurosarcoidosis may be at an increased risk for chronic or recurrent disease. This case highlights the importance of considering sarcoidosis in the differential diagnosis of patients presenting with nonspecific symptoms and lymphadenopathy, even in the absence of pulmonary involvement.

## Introduction

Sarcoidosis is a rare and complex multisystem disease characterized by the formation of noncaseating granulomas in various organs, including the lungs, lymph nodes, skin, eyes, and liver [1]. Clinical presentation of sarcoidosis is variable and can range from asymptomatic to severe, depending on the extent and severity of organ involvement [1]. Skeletal involvement in sarcoidosis is uncommon and occurs in 5-10% of cases [2]. Skeletal manifestations can be the primary or secondary feature of sarcoidosis, and the clinical presentation varies widely depending on the affected bones [3]. The diagnosis of sarcoidosis requires the presence of granulomatous inflammation on histological examination and the exclusion of other causes of granulomatous disease [4]. Here, we present a rare case of a 39-year-old man whose symptoms initially raised concerns for malignancy but were instead the result of sarcoidosis primarily affecting his skeletal system.

## Case presentation

A 39-year-old man presented to our institution’s emergency department with a one-week history of intractable nausea, vomiting, inability to tolerate oral intake, and progressively worsening fatigue. These symptoms were preceded by several months of dull lower back and pelvic pain and a year of unintentional weight loss. His medical history was remarkable for neurosarcoidosis, a seizure disorder on valproic acid, and a benign intracranial granuloma post-surgical resection.

Physical examination revealed dry mucous membranes and left axillary and inguinal lymphadenopathy. Initial laboratory studies revealed normocytic anemia with a hemoglobin level of 9.7, decreased albumin of 2.20 g/dL, and abnormally low serum iron and total iron-binding capacity levels, although his serum ferritin was elevated.

**Figure 1:**
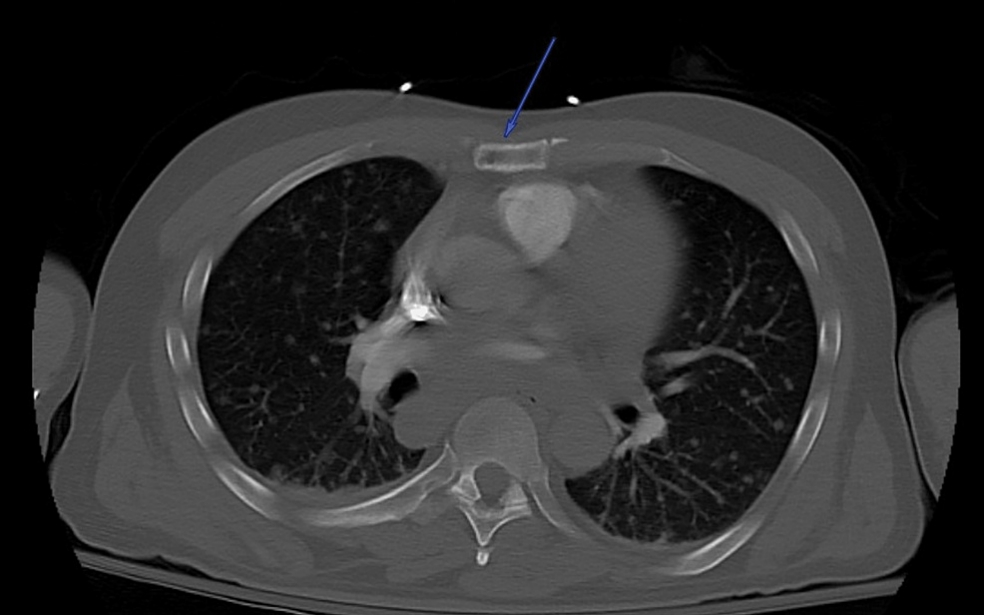
Axial bone window CTA scan of the chest revealing a lytic sternal lesion (blue arrow) CTA: computed tomography angiography

**Figure 2:**
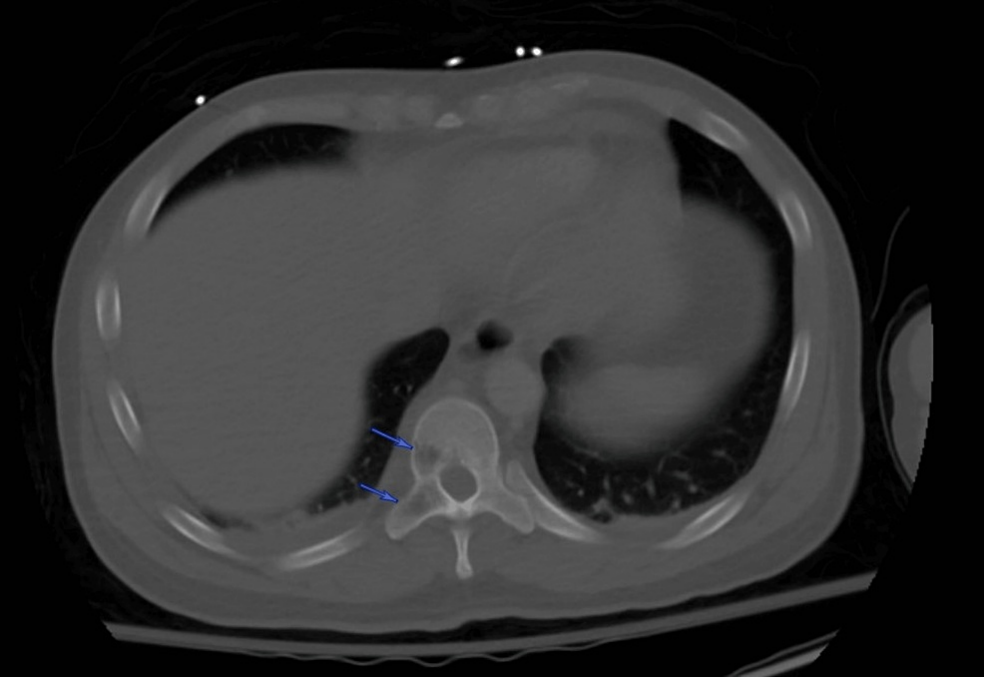
Axial bone window CT scan of the chest with intravenous contrast revealing lytic T10 vertebral lesions (blue arrows) CT: computed tomography

**Figure 3:**
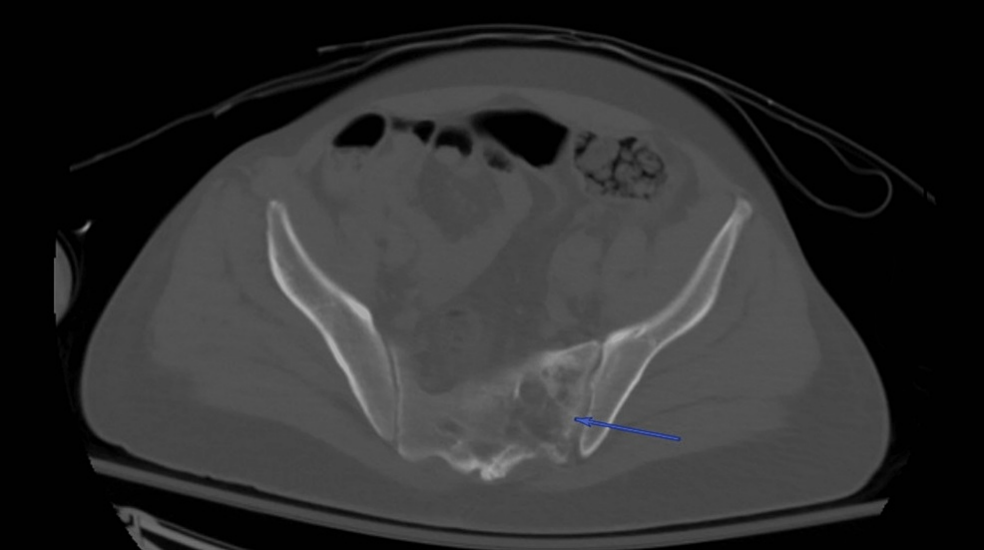
Axial bone window CT scan of the pelvis without intravenous contrast revealing lytic sacral lesions (blue arrow) CT: computed tomography

**Figure 4:**
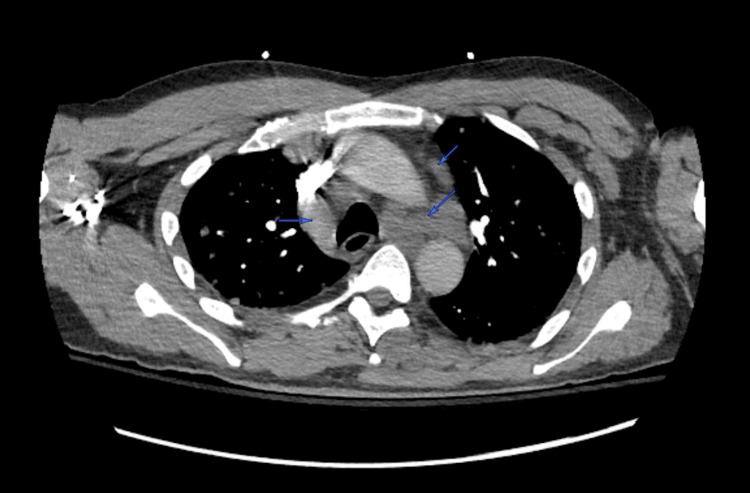
Axial CTA scan of the chest revealing mediastinal, left hilar, and aortocaval lymphadenopathy (blue arrows) CTA: computed tomography angiography

**Figure 5:**
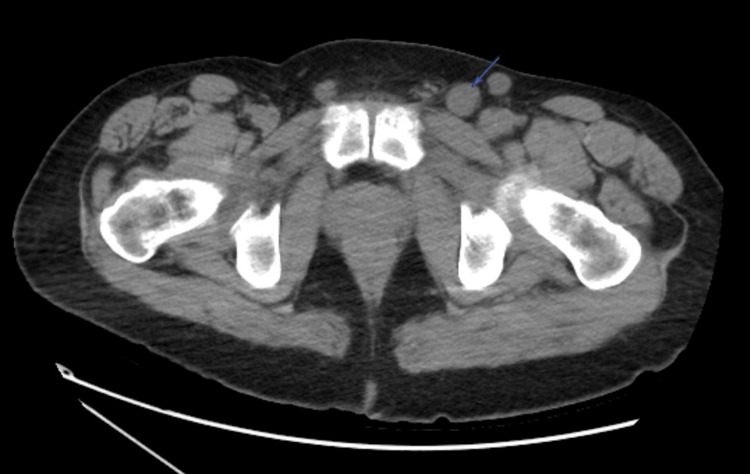
Axial CT scan of the pelvis without intravenous contrast revealing left inguinal lymphadenopathy (blue arrow) CT: computed tomography

Based on these findings, computed tomography (CT) scans of the chest, abdomen, and pelvis were obtained. The images revealed lytic lesions in the sternum (Figure 1), left glenohumeral joint, thoracic (Figure 2) and lumbar spine, and left sacrum (Figure 3). Marked lymphadenopathy was noted in the supraclavicular, mediastinal (Figure 4), left hilar (Figure 4), aortocaval (Figure 4), retroperitoneal common iliac chain, and left inguinal (Figure 5) lymph nodes. Additionally, numerous sub-centimeter, noncalcified pulmonary nodules were seen. These findings raised concerns for metastatic malignancy or lymphoma, so further laboratory testing and a left inguinal lymph node excisional biopsy were performed.

Protein and immunoglobulin (Ig) studies revealed normal total protein but low albumin and elevated alpha-1-, alpha-2-, and gamma-globulins (Table 1). Further immunologic studies showed elevated IgG, IgA, kappa, and lambda light chains with a normal Ig kappa/lambda ratio (Table 1). Protein electrophoresis and immunoelectrophoresis did not detect monoclonal immunoglobulins.

**Table 1:** Laboratory studies *Abnormal values

Laboratory Study	Result	Reference Range
Total Protein	7.3 g/dL	5.5-8.7 g/dL
Albumin	2.20 g/dL*	3.57-5.42 g/dL
Globulin	5.1 g/dL*	2.3-3.5 g/dL
Alpha-1-Globulins	0.45 g/dL*	0.19-0.40 g/dL
Alpha-2-Globulins	1.42 g/dL*	0.45-0.97 g/dL
Beta Globulins	0.99 g/dL	0.54-1.07 g/dL
Gamma Globulins	2.42 g/dL*	0.71-1.54 g/dL
Immunoglobulin G	2444 mg/dL*	700-1600 mg/dL
Immunoglobulin A	643 mg/dL*	70-400 mg/dL
Immunoglobulin M	82 mg/dL	40-230 mg/dL
Immunofixation Kappa Light Chain	75.6 mg/L	3.3-19.4 mg/L
Free Lambda Light Chains	61.3 mg/L*	5.7-26.3 mg/L
Immunoglobulin Kappa/Lambda Ratio	1.23	0.26-1.65

The histopathology of the left inguinal lymph node revealed necrotizing granulomatous lymphadenitis with stellate microabscesses. There was no evidence of lymphoma or metastatic cancer. Acid-fast bacilli and Grocott's methenamine silver stains were negative for acid-fast bacilli and fungal organisms, respectively. Additionally, the patient reported no personal or family history of tuberculosis. These findings suggested that the patient's symptoms were due to sarcoidosis. The patient was treated with intravenous corticosteroids followed by oral steroids, and his condition significantly improved over 11 days. A repeat CT scan of the abdomen and pelvis without intravenous contrast was performed 1.5 months following treatment completion, revealing stable lytic bone lesions, pelvic and inguinal lymphadenopathy, and bilateral pulmonary nodules.

## Discussion

Sarcoidosis is a chronic multisystem disorder characterized by noncaseating granuloma formation, which can affect any organ, with the lungs and lymph nodes being the most commonly involved [1]. Skeletal sarcoidosis is a rare manifestation of the disease, and its prevalence is not well established [5]. Most patients without systemic involvement are asymptomatic on initial presentation, and imaging studies are not routinely performed [5]. Therefore, in these cases, skeletal sarcoidosis is usually incidentally identified on imaging [5].

This patient's symptoms were initially concerning for malignancy; however, he had a history of known neurosarcoidosis, which raised concerns that his presenting symptoms may have been related to sarcoidosis. Additionally, his presentation was consistent with systemic sarcoidosis, which can manifest with a range of symptoms, including fatigue, weight loss, and lymphadenopathy [1]. The findings of his iron studies were consistent with anemia of chronic disease, which is common in sarcoidosis. The presence of lytic bone lesions is less common but can occur in up to 5% of cases [2]. The diagnosis of sarcoidosis is frequently aided by the presence of granulomatous inflammation on histological examination and requires the exclusion of other causes of granulomatous disease (e.g., infection, malignancy) [6]. In this case, the biopsy of the left inguinal lymph node revealed necrotizing granulomatous lymphadenitis, which, when considered with the remainder of the clinical picture, was consistent with necrotizing sarcoid granulomatosis - a rare variant of sarcoidosis with few cases reported to date [7].

Elevation of gamma-globulins in sarcoidosis is a well-known finding, and elevated levels of IgG and IgA are commonly seen in over half and about a quarter of cases, respectively [6,8]. Polyclonal elevations of serum immunoglobulins are consistent with sarcoidosis and suggested that monoclonal gammopathy was unlikely [8].

Systemic corticosteroids are the mainstay of treatment for sarcoidosis [1]. However, the decision to initiate corticosteroids should be made on a case-by-case basis, considering the severity of symptoms, organ involvement, and potential complications of treatment [9]. Given the rarity of skeletal sarcoidosis, standard treatment regimens are not well established; however, about half of the reported cases have shown improvement without treatment [10,11]. If treatment is initiated, systemic corticosteroids, hydroxychloroquine, and methotrexate are most frequently used [10]. Steroid therapy typically consists of oral prednisone starting at 20-40 mg daily with dose reduction after 6-12 weeks [10]. In cases of corticosteroid resistance, combination therapy, including the use of steroid-sparing agents (e.g., hydroxychloroquine, methotrexate, cyclosporine, and tumor necrosis factor inhibitors), may be effective [10]. In this case, the patient was treated with systemic corticosteroids, which resulted in significant clinical improvement.

The prognosis of sarcoidosis is generally good, with spontaneous remission occurring in up to two-thirds of patients [6]. However, some patients can develop chronic or progressive diseases, which can result in significant morbidity and mortality [1]. In particular, patients with a history of neurosarcoidosis may be at an increased risk for chronic or recurrent disease, as was true in this case [12]. For patients with skeletal sarcoidosis, clinical remission and pain improvement have been observed with prednisone and methotrexate [10]. Additionally, radiographic resolution of osseous lesions has been observed with tumor necrosis factor inhibitor therapy [11].

## Conclusions

Overall, this case highlights the importance of considering sarcoidosis in patients with a history of the disease and presenting with nonspecific symptoms such as fatigue, weight loss, and lymphadenopathy. Appropriate diagnostic testing, including biopsies, laboratory studies, and imaging, is crucial for establishing the diagnosis and excluding other causes of granulomatous disease. This case was particularly significant as the primary organ system involved was the skeleton, and the histopathological findings were consistent with necrotizing sarcoid granulomatosis. The management of sarcoidosis requires an individualized approach based on the severity of symptoms, treatment resistance, and potential complications of treatment.
